# HIV envelope tail truncation confers resistance to SERINC5 restriction

**DOI:** 10.1073/pnas.2101450118

**Published:** 2021-05-17

**Authors:** Tafhima Haider, Xenia Snetkov, Clare Jolly

**Affiliations:** ^a^Division of Infection and Immunity, University College London, WC1E 6BT London, United Kingdom

**Keywords:** HIV, Env, SERINC, IFITM, restriction

## Abstract

SERINC5 is a potent lentiviral restriction factor that gets incorporated into nascent virions and inhibits viral fusion and infectivity. The envelope glycoprotein (Env) is a key determinant for SERINC restriction, but many aspects of this relationship remain incompletely understood, and the mechanism of SERINC5 restriction remains unresolved. Here, we have used mutants of HIV-1 and HIV-2 to show that truncation of the Env cytoplasmic tail (ΔCT) confers complete resistance of both viruses to SERINC5 and SERINC3 restriction. Critically, fusion of HIV-1 ΔCT virus was not inhibited by SERINC5 incorporation into virions, providing a mechanism to explain how EnvCT truncation allows escape from restriction. Neutralization and inhibitor assays showed ΔCT viruses have an altered Env conformation and fusion kinetics, suggesting that EnvCT truncation dysregulates the processivity of entry, in turn allowing Env to escape targeting by SERINC5. Furthermore, HIV-1 and HIV-2 ΔCT viruses were also resistant to IFITMs, another entry-targeting family of restriction factors. Notably, while the EnvCT is essential for Env incorporation into HIV-1 virions and spreading infection in T cells, HIV-2 does not require the EnvCT. Here, we reveal a mechanism by which human lentiviruses can evade two potent Env-targeting restriction factors but show key differences in the capacity of HIV-1 and HIV-2 to exploit this. Taken together, this study provides insights into the interplay between HIV and entry-targeting restriction factors, revealing viral plasticity toward mechanisms of escape and a key role for the long lentiviral EnvCT in regulating these processes.

## Significance

SERINCs and IFITMs are potent lentiviral restriction factors that inhibit infection by blocking viral entry into cells. Specifically, SERINCs inhibit viral fusion by targeting the envelope glycoprotein (Env) by a mechanism that remains incompletely understood. Here, we report that truncation of the Env cytoplasmic tail (CT) of HIV-1 and HIV-2 confers complete resistance to SERINC and IFITM restriction. Notably, while the EnvCT of HIV-1 is necessary for viral replication in T cells, HIV-2 does not require the EnvCT. Our data suggest a mechanism by which human lentiviruses can evade restriction that is mediated by the EnvCT but reveal key differences in the likely fitness cost imposed by this on pandemic HIV-1 and nonpandemic HIV-2.

The evolutionary arms race between human immunodeficiency virus type-1 (HIV-1) and host has allowed for efficient viral replication and transmission. Host cells are naturally hostile environments for invading pathogens and have evolved to express an arsenal of antiviral proteins, collectively termed restriction factors that target different steps of the viral life cycle to block replication. In response, viruses including pandemic HIV-1 and related lentiviruses have evolved to evade or directly antagonize host restriction factors, often by encoding viral accessory proteins (reviewed in refs. 1 and 2).

The most recently described family of lentiviral restriction factors are the Serine Incorporator proteins (SERINCs) (3, 4). HIV-1 is most potently restricted by SERINC5 and to a lesser extent by SERINC3 (3, 4). In addition to human and simian lentiviruses, SERINC5 has broad antiviral activity against other retroviruses, including equine infectious anemia virus (5, 6) and murine leukemia virus (3, 4). SERINC5 is composed of 10 transmembrane domains and is primarily localized at the plasma membrane (7) where it gets incorporated into budding virions and inhibits viral entry (3, 4, 8). The lentiviral accessory protein Nef antagonizes SERINC5, excluding it from incorporation into viral particles (3, 4, 8) by removing SERINC5 from the plasma membrane via AP-2–dependent endocytosis (9). This relocalizes SERINC5 to the endosomal pathway, particularly into Rab5^+^ early endosomes, Rab7^+^ late endosomes, and eventually LAMP1+ compartments, for subsequent lysosomal degradation (9).

Although the exact mechanism of how SERINC5 restricts lentiviruses remains incompletely understood, it is clear that the envelope glycoprotein (Env) is crucial in determining sensitivity to SERINC5 (8, 10–12). Env is a heterotrimeric structure composed of gp120 (receptor-binding subunit) and gp41, which contains an extracellular domain that includes the fusion peptide, a transmembrane domain, and a long but largely enigmatic 150-amino–acid cytoplasmic tail (reviewed in ref. 13). HIV-1 enters target cells by fusing with the host cell plasma membrane after the gp120 subunit engages entry receptors CD4 and coreceptor, either CXCR4 or CCR5. Biochemical studies suggest SERINC5 acts to inhibit small fusion pore formation, thereby reducing the efficiency of this fusion process (8), but whether SERINC5 directly interacts with Env to inhibit fusion, or whether indirect effects mediate restriction remains an open question (8, 11, 12). It has been proposed that SERINC5 extracellular loops 3 and 5 potentially interact with the membrane-proximal external region (MPER) of Env gp41 to mediate restriction (7). By contrast, a recent study investigating the effect of SERINC5 on Env clustering on virions indicated that SERINC5 clusters do not colocalize with Env clusters in the viral membrane (11), questioning the contribution of direct interactions to the restriction mechanism.

The HIV-1 Env cytoplasmic tail (EnvCT) is crucial for Env trafficking to sites of virus assembly at the plasma membrane and for incorporation into virions, most importantly during viral replication in CD4+ T cells that are the main targets for HIV-1 in vivo (13–15). The membrane-proximal N terminus of the EnvCT contains a YxxL endocytic motif that binds the clathrin adaptor AP-2 and a C-terminal dileucine motif (16–19) that both act to limit the amount of Env present on the surface of infected cells. While the EnvCT clearly plays an important role in HIV-1 replication and virion morphogenesis, many aspects of the biology of this region of Env remain unclear. Most notably, why lentiviruses like HIV-1 require such a long EnvCT compared to related retroviruses that do not. Interestingly, the majority of the EnvCT is believed to be embedded within the plasma membrane (20) similar to SERINCs (7), where the EnvCT has been shown to influence Env mobility in membranes (21, 22). However, whether this critical region of Env interacts with SERINC5 and what role the EnvCT plays in SERINC5-mediated restriction remains unknown.

Here, we examined the role of the EnvCT in determining sensitivity to SERINC5-mediated restriction with the goal of better understanding the complex relationship between Env and SERINC5. Using replication-competent viral mutants, we show that EnvCT truncation (ΔCT virus) renders both HIV-1 and HIV-2 resistant to SERINC5 and SERINC3 inhibition of infection, despite SERINC being incorporated into viral particles. Further analysis revealed that EnvCT truncation allowed HIV-1 to overcome the SERINC5-mediated block to viral fusion. Truncation of the EnvCT altered Env conformation and functionality, including fusogenicity, thermostability, and entry kinetics, all of which likely contribute to SERINC5 evasion. Furthermore, EnvCT truncation also rendered HIV-1 and HIV-2 viruses resistant to IFITM1, another entry-targeting restriction factor, suggesting an overlap between IFITM and SERINC restriction mechanism and viral evasion. Notably, while HIV-1 cannot replicate in T cells without the EnvCT, HIV-2 viruses are able to replicate efficiently, highlighting important differences in the requirement for the long EnvCT between pandemic HIV-1 and nonpandemic HIV-2. Our results suggest a model in which truncation of the cytoplasmic tail dysregulates Env conformation and functionality, allowing evasion from entry-targeting restriction factors, providing insights into the biology of the EnvCT, specifically its role in innate immune evasion and lentiviral replication.

## Results

### Truncating the HIV EnvCT Overcomes SERINC5 Restriction.

**Fig. 1.**
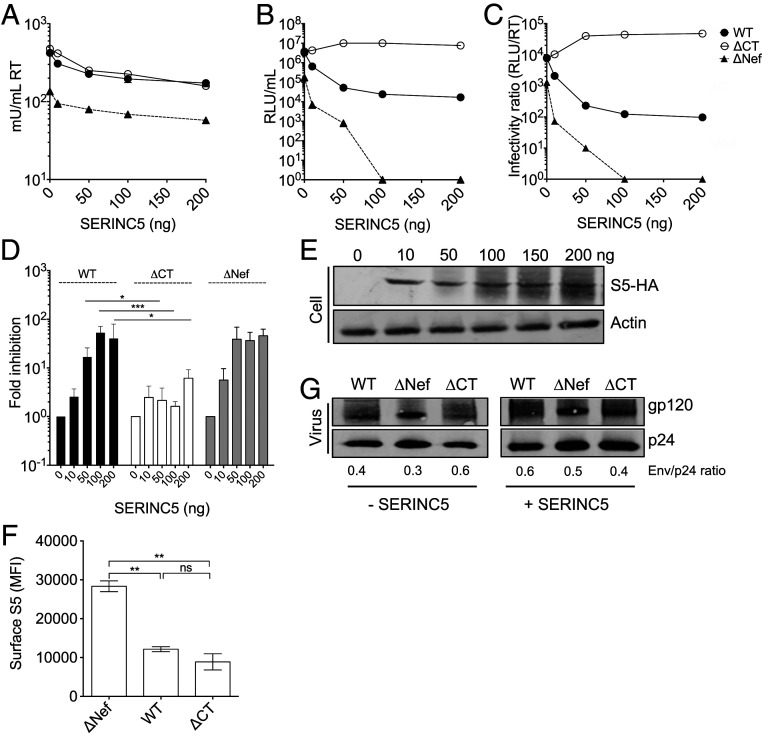
Truncating the HIV-1 EnvCT confers resistance to SERINC5 restriction. 293T cells were cotransfected with full-length HIV-1 NL4.3- WT, ΔNef-, or ΔCT-encoding plasmids alongside SERINC5 plasmid (Flag-SERINC5-HA). Virus-containing supernatants were harvested after 48 h. (*A*) Budding was quantified by RT activity using SG-PERT assay. (*B*) Infectivity was measured on HeLa TZMbl cells by luciferase assay (RLU). (*C*) Particle infectivity was calculated by normalizing infectivity RLU (*B*) to RT activity (*A*). (*A*–*C*) show a representative experiment. (*D*) Fold inhibition of viral infectivity was calculated by normalizing RLU/RT to 0 ng SERINC5 (*n* = 3 independent experiments). HIV-1 WT (black), ΔCT (white), and ΔNef (gray). (*E*) Representative immunoblot of transfected 293T cells probed for HA-tagged SERINC5 (S5-HA). (*F*) Plasma membrane SERINC5 levels on transfected 293T cells stained for an external Flag-tagged and intracellular HA-tagged SERINC5. The surface SERINC5 mean fluorescent intensity is shown for cells gated on HA-SERINC5 and Gag. (*G*) Immunoblot of purified virions produced in the presence and absence of overexpressed SERINC5, probed for Envgp120 and Gagp24. The ratio of Env incorporation calculated by measuring the intensity of gp120 normalized to corresponding Gagp24. The bars show mean ± SEM. Fold inhibition at each dose of SERINC5 was compared using two-way ANOVA testing (not significant [ns], *P* > 0.05; *P < 0.05; **P < 0.01; ***P < 0.001).

To investigate the role of the EnvCT in SERINC5-mediated restriction, we generated a panel of mutant HIV viruses with a stop codon in the EnvCT downstream of the Y_712_xxL endocytic motif (termed ΔCT viruses) (*SI Appendix*, Fig. S1). For HIV-1 NL4.3, this stop codon was inserted at Env position 722. For HIV-2 ST and 7312A, stop codons were inserted at position 713 (*SI Appendix*, Fig. S1), corresponding to where HIV-2 and several Simian immunodeficiency viruses (SIVs) have been reported to naturally truncate the EnvCT to gain a replicative advantage during in vitro passage in human PBMCs and T cell lines (23–31). To determine the consequence of EnvCT deletion for SERINC5 restriction, we used an established cotransfection assay to interrogate SERINC5 restriction (3, 4, 8, 10). 293T cells were cotransfected with replication-competent HIV-1 or HIV-2 molecular clones encoding either a full-length (WT) or truncated (ΔCT) EnvCT alongside increasing doses of plasmid encoding SERINC5, allowing overexpression of SERINC5 in producer cells. Of note, 293T cells are permissive for EnvCT deletion (15, 32), providing a tractable model to interrogate specific aspects of EnvCT biology. At 48 h post-transfection, supernatants were harvested, and viral budding was quantified using SYBR Green I–based product-enhanced RT (SG-PERT) assay (33) for RT activity (Fig. 1*A*) and infectivity quantified by titrating virus-containing supernatants onto HeLa TZMbl luciferase reporter cells (Fig. 1*B*). Normalizing infectivity (RLU) to budding (RT activity) was used to calculate particle infectivity (RLU/RT) (Fig. 1*C*). As expected, the infectivity of HIV-1 ΔNef virus (that cannot antagonize SERINC5) was potently restricted in a dose-dependent manner when virions were produced in the presence of SERINC5 (Fig. 1 *B*–*D*) consistent with previous reports (3, 4, 8, 10). By contrast, HIV-1 WT, which encodes of a functional Nef protein, was significantly less restricted (Fig. 1 *B*–*D*), indicative of Nef-mediated SERINC5 antagonism. Although consistent with previous reports (3, 4), in this assay, SERINC5 was capable of inhibiting WT virus due to SERINC5 overexpression overwhelming Nef activity. The reduced infectivity of ΔNef compared to WT virus in the absence of overexpressed SERINC5 (0 ng) is due to endogenous SERINCs expressed by 293T cells (3, 4). Strikingly, EnvCT truncation rendered HIV-1 ΔCT completely resistant to SERINC5 restriction (Fig. 1 *B*–*D*). Even at the highest dose where we observed a 100-fold reduction in WT infection, the infectivity of ΔCT virus remained unaffected (Fig. 1 *C* and *D*). Western blotting cell lysates confirmed SERINC5 overexpression (Fig. 1*E*). Flow cytometry analysis of cell-surface SERINC5 (stained for an external FLAG-tag) confirmed both HIV-1 WT and ΔCT viruses similarly down-regulated SERINC5, as expected since both viruses contain a *Nef* allele (Fig. 1*F*). Similar results were obtained for HIV-1 WT and ΔCT virus in the presence of increasing doses of SERINC3, with ΔCT virus completely resistant to SERINC3 restriction (*SI Appendix*, Fig. S2*A*). As previously reported (3, 4), SERINC3 was a less-potent inhibitor of HIV-1 infectivity compared to SERINC5.

It has been previously shown that truncating the HIV-1 EnvCT, particularly removing the YxxL endocytic motif and the C-terminal dileucines (commonly referred to as Δ144 virus), can result in increased surface expression of Env due to reduced endocytosis (13, 18, 19, 34) that may impact Env incorporation into nascent virions. Therefore, we tested if our HIV-1 EnvCT truncation mutant (which retains the YxxL but not the C-terminal endocytic motif) showed increased Env incorporation into virions that might explain its ability to overcome SERINC5 restriction. Fig. 1*G* shows both HIV-1 WT and ΔCT viruses produced by 293T cells incorporated similar levels of Env in the presence and absence of SERINC5. From these data, we conclude that resistance of HIV-1 ΔCT virus to SERINC5 is not due to increased Env incorporation by these mutants.

**Fig. 2.**
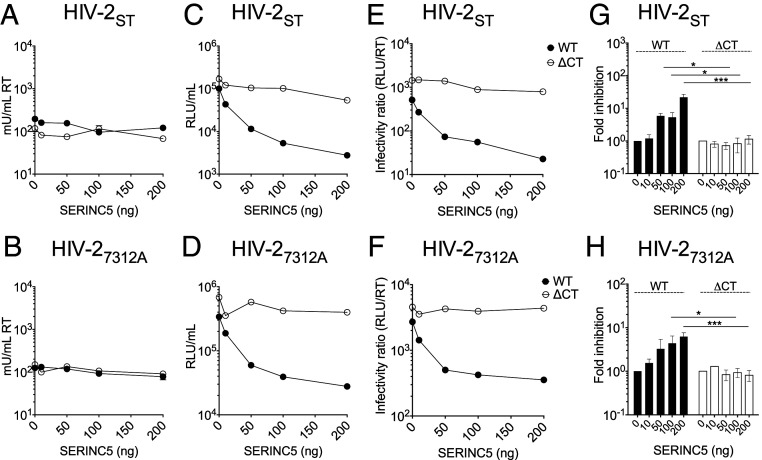
Truncating the HIV-2 EnvCT confers resistance to SERINC5 restriction. 293T cells were cotransfected with full-length HIV-2 WT- and ΔCT-encoding plasmids alongside SERINC5 plasmid (Flag-SERINC5-HA). Virus-containing supernatants were harvested after 48 h. (*A* and *B*) Budding was quantified by RT activity using SG-PERT assay. (*C* and *D*) Infectivity was measured on HeLa TZMbl cells by luciferase assay (RLU). (*E* and *F*) Particle infectivity was calculated by normalizing infectivity RLU to RT activity. These data are from a representative experiment. (*G* and *H*) Fold inhibition of viral infectivity calculated by normalizing RLU/RT to 0 ng SERINC5 (*n* = 3 independent experiments). The bars show mean ± SEM. Fold inhibition at each dose of SERINC5 was compared using two-tailed unpaired *t* test (*P < 0.05; ***P < 0.001).

Next, we tested whether truncation of the HIV-2 EnvCT also conferred resistance to SERINC5 restriction. HIV-2 Nef is reportedly less active against SERINC5 than HIV-1 (35); however, this was performed in the context of chimeric HIV-1 NL4.3 encoding a HIV-2 *Nef* allele rather than full-length HIV-2 virus. Fig. 2 shows HIV-2 strains ST and 7312A were both restricted up to 10-fold when produced in the presence of overexpressed SERINC5, whereas HIV-2 ΔCT viruses remained fully infectious even at high doses of SERINC5 (Fig. 2). By contrast to SERINC5, SERINC3 did not restrict HIV-2, with both 7312A and ST viruses showing no loss of virion infectivity (*SI Appendix*, Fig. S2 *B*–*D*).

### EnvCT Truncation Overcomes SERINC5-Mediated Inhibition of Fusion.

**Fig. 3.**
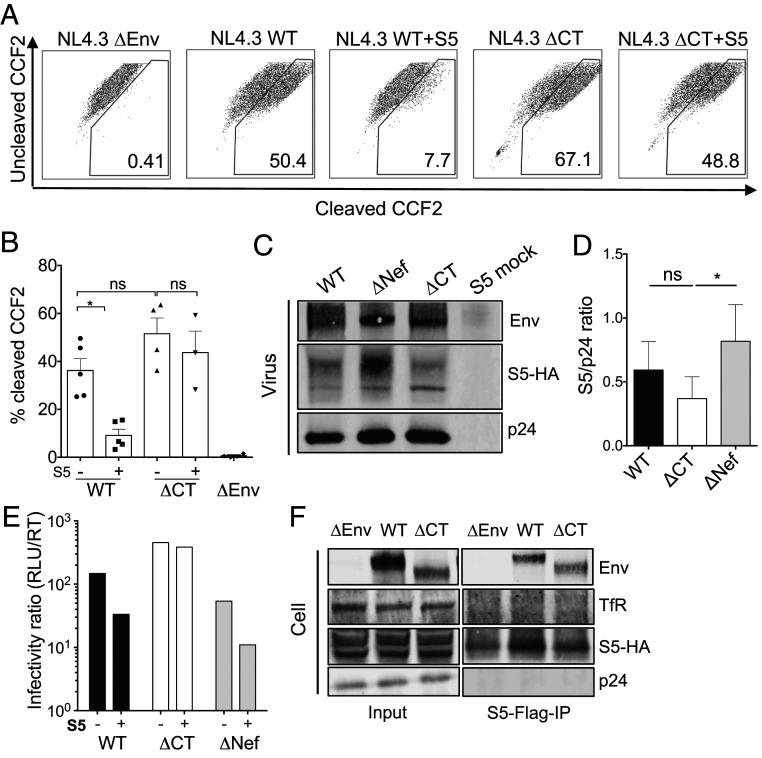
EnvCT truncation allows evasion of SERINC5-mediated inhibition of fusion. Viral fusion measured by Blam-Vpr assay on HeLa TZMbl cells using HIV-1 WT and ΔCT virions made in the presence or absence of 100 ng SERINC5. (*A*) Representative flow cytometry dot plots indicating percentage of CCF2 substrate cleavage. (*B*) Pooled data from five independent experiments. ΔEnv virus is a negative control. (*C*) Immunoblot analysis of purified virions probed for Envgp120, HA-tagged SERINC5 (S5-HA), and Gagp24. Supernatant from 293T cells transfected with SERINC5 plasmid only was purified and probed for SERINC5 as a control (S5 mock). (*D*) Quantification of SERINC5 incorporation normalized to Gagp24 from five independent experiments. (*E*) Infectivity of virions from the corresponding immunoblot in *C*. (*F*) 293T cells cotransfected with FLAG-tagged SERINC5 (Flag-S5-HA) and plasmid encoding HIV-1 WT, ΔCT, or ΔEnv virus. Flag-SERINC5 was immunoprecipitated and immunoblot probed for HA-tagged SERINC5, Envgp120, Transferrin receptor (TfR) and Gagp24. A representative immunoblot is shown. The bars represent mean ± SEM. The groups were compared using two-way ANOVA multiple comparison analysis (not significant [ns], *P* > 0.05; *P < 0.05).

SERINC5 is thought to inhibit HIV-1 infection by targeting the step of fusion between the viral lipid envelope and the host cell plasma membrane (4, 8, 36). Therefore, we tested whether EnvCT truncation allowed HIV-1 to evade SERINC5 inhibition of virion fusion using the Blam-Vpr fusion assay (37). Consistent with the notion that SERINC5 targets viral fusion, a significant 6.5-fold reduction in fusion of HIV-1 WT virus was observed when virions were produced in the presence of SERINC5 (Fig. 3 *A* and *B*). Notably, fusion of the HIV-1 ΔCT Env was unaffected by SERINC5 overexpression (Fig. 3 *A* and *B*). These data reveal a mechanism for HIV-1 ΔCT Env overcoming SERINC5-mediated inhibition at the step of viral fusion.

It has been suggested that incorporation of SERINC5 into virions is likely necessary to inhibit HIV-1 infectivity (3, 4, 10, 12). To test whether HIV-1ΔCT virus incorporates SERINC5, Western blotting was performed on virus produced in the presence of SERINC5 (Fig. 3 *C* and *D*). We used a dose of SERINC5 plasmid (100 ng) that inhibits HIV-1 WT and ΔNef but not ΔCT infectivity (Fig. 3*E*). Both HIV-1 WT and ΔCT viruses incorporated SERINC5 into virions to similar levels (Fig. 3 *C* and *D*). As expected, ΔNef virus (that is unable to downmodulate SERINC5 from the plasma membrane) incorporated more SERINC5 into virions (Fig. 3 *C* and *D*). Therefore, the resistance of HIV-1 ΔCT virus to SERINC5 restriction of virion fusion cannot be explained by failure to package SERINC5 into nascent virions, supporting the notion that SERINC5 incorporation may be necessary but is not sufficient for restriction.

### EnvCT Truncation Does Not Prevent Potential SERINC5–Env Interactions.

Whether SERINC5 and Env physically interact on the plasma membrane of infected cells or in virions remains unclear (8, 11, 12) but has important implications for understanding mechanism of SERINC5 restriction and viral evasion. To investigate whether SERINC5 binds to HIV-1 Env, and if so whether this is EnvCT dependent, we performed a coimmunoprecipitation (Co-IP) assay. 293T cells were cotransfected with Flag-tagged SERINC5 and plasmid encoding either HIV-1 WT, ΔCT, or ΔEnv, and SERINC5 was pulled down using α-FLAG antibody. Fig. 3*F* shows that both the WT and ΔCT Env glycoproteins coimmunoprecipitated with SERINC5. As expected, no band was detected using α-gp120 serum in the ΔEnv condition (Fig. 3*F*). As an additional control, we also probed for two proteins that are not expected to interact with SERINC5, the transferrin receptor and the HIV-1 Gag protein, both of which failed to co-IP with SERINC5 (Fig. 3*F*). Taken together, these data are suggestive of an interaction between SERINC5 and Env mediated by domains upstream of residue 722 in the EnvCT. Furthermore, evasion of SERINC5 restriction by ΔCT cannot be explained by a loss of any putative interaction between Env and SERINC5.

### EnvCT Truncation Dysregulates Env Conformation and Functionality.

**Fig. 4.**
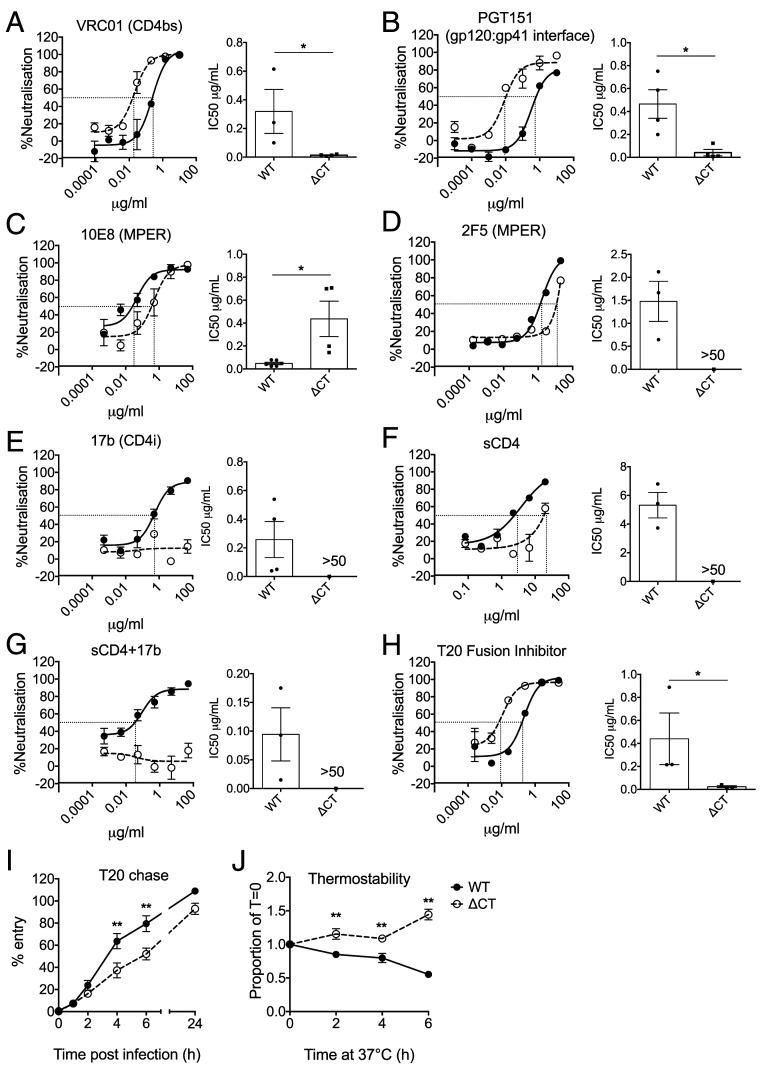
EnvCT truncation alters Env conformation and functionality. (*A*–*G*) Neutralization of HIV-1 WT (solid lines, black symbols) and ΔCT viruses (dotted lines, white symbols) by bnAbs. Representative neutralization curves are shown. The bar charts show mean IC_50_ values from pooled independent experiments. (*H*) A representative T20 neutralization curve is shown; the bar chart shows IC_50_ values from four independent experiments. (*I*) T20 chase assay. Virus was added to HeLa TZMbl cells and T20 added after indicated times. Infection was measured after 48 h by luciferase assay. The data are shown as percentage virus entry normalized to untreated (no T20) control from three independent experiments. (*J*) Thermostability of Env measured by incubating virus at 37 °C for indicated time periods prior to infection of HeLa TZMbl cells. Infection was measured after 48 h by luciferase assay. The data are from three independent experiments. The bars represent mean ± SEM. The groups were compared using two-tailed unpaired Student’s *t* tests (*P* > 0.05; *P < 0.05; **P < 0.01).

Our results demonstrating EnvCT truncation allows for virion fusion in the presence of SERINC5 suggests that truncation of the cytoplasmic tail confers functional changes on extracellular domains of Env. This suggests a mechanism of evasion in which EnvCT truncation alters the conformation of Env, dysregulating the steps of viral entry such that SERINC5 cannot inhibit this process. Therefore, we investigated whether changes in the conformation of HIV-1 ΔCT Env mutant were associated with escape from SERINC5 restriction of viral fusion by our mutants. Neutralization assays were performed using a panel of well-characterized broadly neutralizing antibodies (bnAbs) against HIV-1. Neutralization curves for VRC01 (CD4 binding site) (38) and PGT151 (gp120:gp41 interface and functional trimers) (39) revealed that ΔCT virus was more susceptible to neutralization by these gp120-targeting antibodies (half maximal inhibitory concentration (IC_50_) ΔCT 0.02 and 0.04 μg/mL for VRC01 and PGT151, respectively) compared to WT virus (IC_50_ WT 0.31 and 0.46 μg/mL for VRC01 and PGT151, respectively) (Fig. 4 *A* and *B*). Conversely, ΔCT viruses were significantly more resistant to gp41 MPER–targeting bnAbs 10E8 and 2F5 compared to WT (IC_50_ ΔCT of 0.43 and >50 μg/mL versus WT 0.04 and 1.5 μg/mL) (Fig. 4 *C* and *D*). Incorporation of SERINC5 into WT and ΔCT viruses did not alter sensitivity to VRC01, but WT virus showed a nonsignificant trend toward altered sensitivity to PGT151 (*SI Appendix*, Fig. S3 *A*–*D*). Moreover, we also saw a trend toward altered sensitivity to MPER-targeting antibodies (*SI Appendix*, Fig. S3 *E*–*H*) (4, 7, 8, 10). These results are consistent with the hypothesis that HIV-1 EnvCT truncation alters the conformation of Env, particularly the MPER, allowing evasion from SERINC5.

The bnAb 17b recognizes a CD4-inducible epitope and is a coreceptor-blocking antibody (40). Most strikingly, 17b neutralized HIV-1 WT with an IC_50_ of 0.25μg/mL, by contrast to ΔCT virus which was completely resistant to neutralization (Fig. 4*E*). Preincubation of virus with soluble CD4 (sCD4) is expected to induce conformational changes in gp120 to open Env trimers and further expose cryptic epitopes, including that recognized by 17b. To test if this would sensitize HIV-1 ΔCT to 17b, we first performed a neutralization assay with sCD4 to determine a subinhibitory dose of sCD4, selecting 1μg/mL that resulted in ∼25% neutralization of both viruses (Fig. 4*F*). However, HIV-1 ΔCT virus remained completely resistant to 17b neutralization (Fig. 4*G*). Interestingly, the sCD4 neutralization curves (Fig. 4*F*) suggested that ΔCT Env required more sCD4 for neutralization compared to WT (IC_50_ values > 50 μg/mL and 5.3 μg/mL, respectively), consistent with EnvCT truncation influencing Env conformation and function.

Having shown ΔCT virus is resistant to SERINC5 inhibition of fusion, and EnvCT truncation alters the conformation of Env extracellular domains, we hypothesized that EnvCT truncation may alter the processivity of Env-mediated fusion. To test this, we performed a neutralization assay with the fusion inhibitor T20. Fig. 4*H* shows that ΔCT virus was more sensitive to neutralization by T20 when compared to WT virus (IC_50_ WT 0.4μg/mL versus ΔCT 0.02μg/mL). Next, we measured fusion kinetics of HIV-1 WT and ΔCT virus using a T20 chase assay. To do this, virus was incubated with cells and T20 added at various time points postinfection to determine when entry became resistant to fusion inhibition. Fig. 4*I* shows that by 6 h postaddition of virus, T20 was unable able to inhibit HIV-1 WT fusion and block infection, indicating entry is complete. By contrast, at this same time point, only 50% of ΔCT infection was blocked, indicating that the ΔCT virus has altered fusion and entry kinetics (Fig. 4*I*). By 24 h postinfection, both WT and ΔCT viruses became resistant to T20 inhibition, suggesting that the process of entry was complete. It was intriguing that the HIV-1 ΔCT virus displayed slower fusion kinetics than WT virus during the first 6 h of entry but eventually caught up with completion of entry between 16 (BLAM-Vpr assay, Fig. 3) and 24 h (T20 chase, Fig. 4). To explore this further, we incubated HIV-1 WT and ΔCT viruses at 37 °C for various times and measured Env-dependent viral infectivity. Fig. 4*J* shows ΔCT virus was significantly more stable than WT, the latter showing a time-dependent reduction in thermostability. Taken together, these data suggest HIV-1 EnvCT truncation confers conformational changes on Env that are reflected by altered neutralization and viral fusion kinetics that may collectively contribute to evasion of SERINC5 restriction.

### HIV EnvCT Truncation Also Confers Resistance to IFITM1 Restriction.

**Fig. 5.**
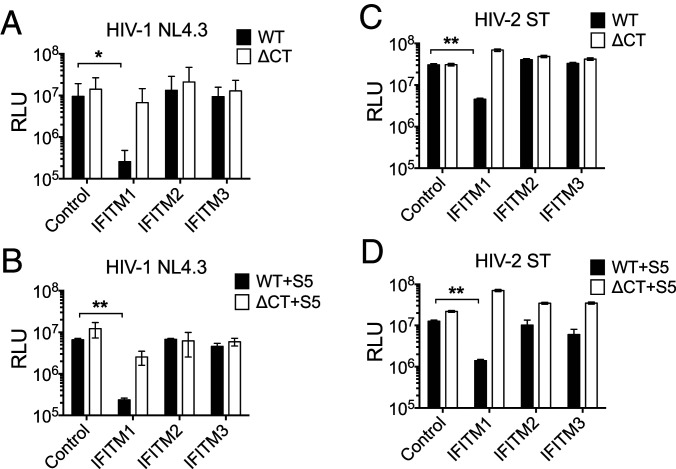
Truncation of the HIV EnvCT confers resistance to IFITM1 restriction. TZMbl cells were transduced with pSIN vectors expressing IFITM1, IFITM2, or IFITM3 and selected using puromycin. WT and ΔCT viruses were made in 293T cells in the presence and absence of 100 ng SERINC5. (*A*–*D*) Control TZMbl cells (no IFITM overexpression) and IFITM-overexpressing cells were infected with equal RT units of virus for 24 h. Infectivity was measured by luciferase assay. The bars show mean, and the error bars represent mean ± SEM from three independent experiments. Infectivity inhibition was compared using two-way ANOVA (*P < 0.05; **P < 0.01).

The IFITM proteins are another potent entry-targeting restriction factor family that can inhibit HIV-1 infection when present in target cells during entry (41–44) and also when expressed in virus-producing cells (45–47). Whether HIV-2 is similarly sensitive to IFITMs is less well understood. Therefore, we investigated the relationship between SERINC5 and IFITM restriction of human lentiviruses and tested whether truncation of the EnvCT also overcomes restriction of viral entry by IFITMs in target cells. To do this, HA-tagged IFITM 1, 2, and 3 were expressed in the HeLa TZMbl reporter cell line using lentiviral transduction (*SI Appendix*, Fig. S4*A*). To validate this assay, we showed that the transmitted/founder virus HIV-1 CH058 was resistant to inhibition by IFITM proteins in target cells as reported previously (*SI Appendix*, Fig. S4*B*) (43), whereas VSV-G pseudotyped HIV-1 ΔEnv virus was restricted by IFITM3 (*SI Appendix*, Fig. S4*C*), also as previously described (42, 44). Next, IFITM-expressing HeLa TZMbl target cells were infected with equal RT units of HIV-1 and HIV-2 WT or ΔCT viruses and infection measured. Fig. 5*A* shows that HIV-1 WT virus was potently restricted up to 50-fold by IFITM1 but not IFITM2/3, the former being plasma membrane localized and the latter mainly located in early and late endosomes (43), consistent with HIV-1 entering by fusion at the plasma membrane. No difference in IFITM inhibition of virus infection of target cells was observed when HIV-1 WT virus was produced in the presence of SERINC5 (Fig. 5*B*), demonstrating that SERINC5 does not alter HIV-1 sensitivity to IFITM proteins in target cells. Notably, HIV-1 ΔCT was completely resistant to IFITM 1, 2, and 3 (Fig. 5*A*), similar to what was observed for SERINCs, and SERINC5 did not sensitize ΔCT virus to IFITM restriction (Fig. 5*B*). Turning to HIV-2, Fig. 5*C* shows that HIV-2 WT was also restricted by IFITM1, and again SERINC5 incorporation did not alter sensitivity of this virus to IFITMs (Fig. 5*D*). Like HIV-1, we found that EnvCT truncation allowed HIV-2 ΔCT virus to evade IFITM1-mediated inhibition (Fig. 5 *C* and *D*).

### HIV-1 and HIV-2 Have a Differential Requirement for the Long EnvCT during Viral Replication.

**Fig. 6.**
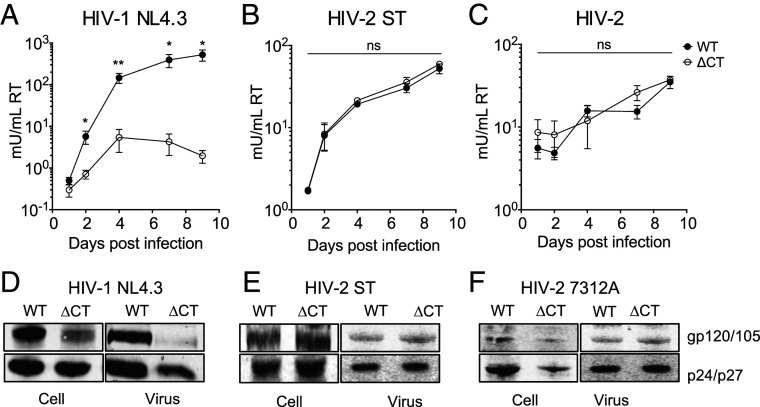
HIV-1 and HIV-2 viruses have a differential requirement for the EnvCT. H9 cells were infected with equal RT units of VSV-G–pseudotyped Env-expressing infectious virus (VSV-G used to normalize initial infection). (*A*–*C*) Supernatants were collected over time and virus quantified by SG-PERT assay for spreading infection of full-length (FL; closed circles) or truncated EnvCT (ΔCT; open circles) viruses. The data from three independent experiments. (*D*–*F*) Representative immunoblots showing Env incorporation. H9 cells infected with HIV-1 NL4.3, HIV-2 ST, or HIV-2 7312A WT and ΔCT virus. Cell lysates and purified virus immunoblots were probed for Envgp120 or gp105 and Gagp24 or p27. The bars show mean ± SEM. Infectivity of WT and ΔCT virus at each time point was compared using the two-tailed unpaired Student’s *t* test (not significant [ns]; *P < 0.05; **P < 0.01).

Given that truncation of the lentiviral EnvCT confers resistance to two potent viral entry-targeting restriction factors, we tested whether this conferred an advantage to viral replication and spreading infection in T cells. H9 T cells [expressing CD4 and both CXCR4 and CCR5 (48)] were infected with VSV-G pseudotyped HIV-1 and HIV-2 WT and ΔCT viruses in order to achieve similar levels of initial infection. To monitor viral replication and spread over time, cell culture supernatants were collected for 9 d, and virus production quantified by SG-PERT. As expected, HIV-1 WT virus with a full-length EnvCT replicated and spread well over time (Fig. 6*A*). By contrast, NL4.3 ΔCT virus failed to replicate and spread as efficiently in H9 cells (Fig. 6*A*), consistent with previous studies showing that the EnvCT is required for HIV-1 replication in most T cell lines and primary T cells (13–15). By contrast, replication of WT and ΔCT HIV-2 strains ST and 7312A were comparable over time, with ΔCT virus showing no defect during spreading infection in T cells (Fig. 6 *B* and *C*), consistent with early studies reporting spontaneous HIV-2 EnvCT truncations in vitro (24, 26, 28). Thus, while HIV-1 requires a long EnvCT for replication in T cells, HIV-2 can replicate in the absence of the majority of the EnvCT. The requirement for the EnvCT for Env incorporation into virions during assembly in T cells in well-established for HIV-1 (13–15); therefore, we also analyzed HIV-2 Env incorporation into virions produced from infected H9 cells. As expected, HIV-1 ΔCT virus incorporated less Env into viral particles compared to WT virus (Fig. 6*D*), in line with previous studies showing that almost-complete removal of the HIV-1 EnvCT (NL4.3 Δ144 mutant) produces viruses with a defect in Env incorporation in T cells (14, 15). Conversely, HIV-2 WT and ΔCT viruses incorporated similar levels of Env (Fig. 6 *E* and *F*), explaining their ability to replicate in T cells (Fig. 6 *B* and *C*).

## Discussion

Here, we have uncovered a mechanism by which HIV-1 and HIV-2 may evade restriction by two potent entry-targeting restriction factors, SERINC3/5 and the IFITMs. For both sets of restriction factors, inhibition of viral entry is mediated at the step of viral fusion and displays an Env-dependent phenotype (reviewed by ref. 2). We find a commonality in the mechanism of HIV evasion from these two families of restriction factors where EnvCT truncation allows HIV-1 and HIV-2 to escape inhibition. SERINC5 restricts viral entry by inhibiting fusion of virions with target cell membranes (3, 4, 8), and we show that truncation of the EnvCT bypasses this block, allowing fusion to take place. Importantly, evasion from restriction was not due to complete exclusion of SERINC5 from ΔCT virus, consistent with the notion that SERINC5 incorporation may be necessary but is not sufficient for restriction of HIV-1 infection. This finding is supported by the identification of SERINC5 mutants which cannot restrict HIV-1 despite being efficiently packaged into virions (7). Furthermore, evasion from fusion inhibition was not simply due to more Env being packaged into virions and overcoming the presence of SERINC5. Instead, our data suggest EnvCT truncation alters the conformation and functionality of Env that disarms SERINC5′s restrictive ability.

Currently, three broad but not mutually exclusive mechanisms for how SERINC5 operates to restrict HIV-1 entry have been proposed: 1) inhibition of fusion pore formation during viral entry into target cells (3, 4, 8); 2) spontaneous inactivation of Env trimers (8); and 3) disruption of Env clustering on membranes (11). Given that viral fusion and entry requires a series of well-orchestrated, sequential Env-dependent events, it is likely that SERINC5 may target any or all of these processes to inhibit the final step in Env-mediated entry, viral fusion. Indeed, the phenotypic and functional differences in Env we observed following EnvCT truncation support a model in which removal of the EnvCT alters the conformation and function of the viral glycoprotein, dysregulating Env and collectively reducing the opportunity for SERINC5 to target different properties of Env that are necessary to mediate entry.

We found that both HIV-1 NL4.3 (X4-tropic) and HIV-2 primary isolates (R5-tropic) were sensitive to SERINC5 overexpression. SERINC5 sensitivity has been mapped to the gp120 variable loops, particularly the V3 loop (4, 10, 12), leading to the suggestion that coreceptor usage may separate SERINC5-sensitive and -resistant viruses (10). However, in light of our results, we propose that coreceptor usage mediated by the V3 loop does not fully explain how V3 determines sensitivity to SERINC5. Rather, our data suggest that other properties of Env, which are regulated by the long EnvCT, determine sensitivity to SERINC5. Specifically, HIV-1 Env can exist in what is referred to as either an “open” or “closed” conformation (reviewed by ref. 49). Whether Env is in an “open” or “closed” conformation is partially determined by the positioning and accessibility of the V3 loop, required for coreceptor binding and initiation of fusion. When Env is in a closed, prefusion state, the V3 loop is occluded by V1/2 so the coreceptor binding site is largely inaccessible (50–53). Our data showing that HIV-1 ΔCT was less sensitive to neutralization by bnAbs, such as 17b, 10E8, and 2F5, which target cryptic epitopes in Env that are transiently exposed when Env trimers adopt an open, fusion-intermediate conformation (54), are consistent with ΔCT Env displaying an altered, potentially more-closed conformation that may in turn influence its ability to be targeted by SERINC5. The relationship between Env conformation and SERINC5 restriction is supported by our results and others showing that SERINC5 incorporation into virions increases sensitivity to MPER-targeting bnAbs (8, 10). In support of the notion that ΔCT Env is conformationally dysregulated, we found HIV-1 ΔCT virus had slower entry kinetics and altered sensitivity to the fusion inhibitor T20 than WT virus despite ultimately achieving fusion. We propose that EnvCT truncation regulates the extracellular domains of Env and alters the requirement for Env to undergo the same series of sequential structural changes to mediate viral entry. This is supported by recent NMR studies providing structural evidence that the EnvCT is physically coupled to the Env ectodomain via the gp41 transmembrane domain and thus influences conformation and antigenicity of Env, particularly the gp41 MPER (55). Indeed, we also found that deletion of the majority EnvCT conferred global conformational changes to the extracellular domains of Env, including the gp41 MPER and gp120 receptor–interacting domains. Importantly, our data showing that EnvCT truncation alters SERINC5 restriction, likely via alterations to the Env ectodomain, are consistent with previous studies showing that EnvCT truncation impacts on HIV and SIV Env ectodomain conformation and thus antigenicity and/or fusion activity (56–70).

Like SERINCs, IFITM-mediated restriction of HIV-1 infectivity occurs at the step of fusion and prevents viral entry into target cells (41). We focused on IFITMs expressed in target cells, although inhibitory activity is reported in both target and producer cells (41–47). Like SERINCs, the exact mechanism of IFITM restriction remains unclear. HIV-1 sensitivity to IFITM1-3 has also been mapped to the gp120 V3 loop (43, 46, 71, 72), and coreceptor usage is reported to determine sensitivity to different IFITM proteins (43). Given the similarities between SERINC5 and IFITM restriction targeting viral entry, we tested our HIV-1 and HIV-2 viruses to determine whether EnvCT truncation also overcomes IFITM restriction and whether SERINC5 incorporation alters sensitivity to IFITMs during viral entry. Notably, EnvCT truncation rendered both HIV-1 and HIV-2 ΔCT viruses resistant to IFITM-mediated restriction, specifically IFITM1. In support of this, EnvCT truncation has been reported to relieve a block imposed by IFITM proteins on cell–cell spread and cell–cell fusion (47). Furthermore, we found that SERINC5 incorporation in virions did not alter sensitivity to inhibition by IFITMs in target cells. Using a panel of HIV-1 T/F and chronic viruses, Foster and colleagues showed that the localization of IFITMs as well as properties of Env both determine sensitivity and specificity to IFITMs (43). Viruses that required the CCR5 coreceptor were more susceptible to inhibition by IFITM1 at the plasma membrane, while CXCR4-using viruses were more sensitive to IFITM2/3 that are predominantly localized within endosomal compartments. Despite HIV-2 ST being an R5-tropic virus, we found it was restricted by IFITM1 but not IFITM2/3, similar to X4-tropic HIV-1 NL4.3. Furthermore, IFITM-resistant viruses have been reported to be less susceptible to sCD4 and 17b neutralization by comparison to IFITM-sensitive viruses (71), in keeping with the observation that ΔCT viruses are less sensitive to sCD4 and 17b neutralization. Inconsistency exists about the hierarchy of IFITM1-3 inhibition of HIV-1, the magnitude of inhibition, and how coreceptor usage dictates differential sensitivity to IFITM1-3 (41–44, 47, 71, 73, 74). This likely reflects a combination of differences in the cell types used (i.e., whether viral entry favors plasma membrane or endosomal fusion and the endocytic capacity of certain cell types), how infection is measured (green fluorescent protein reporter virus, viral protein synthesis, or virion infectivity) and the use of pseudotyped viruses compared to replication competent virus (the latter incorporating native levels of Env into virions and also expressing viral accessory proteins, whereas pseudotyped viruses are made using highly overexpressed Env, resulting in high surface Env levels (55), which may overcome IFITM1 inhibition). We used replication-competent infectious virus and a direct measure of infection by expressing IFITM1-3 in HeLa TZM-bl reporter cells that express HIV-1 Tat–driven luciferase. Having validated this assay using T/F virus CH058 and VSV-G Env, we found that HIV-1 and HIV-2 infection was restricted by IFITM1 but not IFITM2 or 3, consistent with the notion that productive HIV infection is mediated by viral fusion at the plasma membrane. Notably, our data reporting sensitivity of HIV-1 to IFITM1 is supported by previous studies that also observed IFITM1 inhibition of infection/fusion using replication-competent CXCR4-tropic HIV-1 (42–44, 47).

Our results indicate a commonality in how SERINC5 and IFITMs can be evaded by human lentiviruses. How might this be achieved? While conformational changes in Env mediated by EnvCT deletion may similarly allow Env to bypass targeting by IFITMs as discussed earlier for SERINC5, we cannot exclude other mechanisms of evasion may be involved. For example, it has been proposed that SERINC5 rigidifies the viral and target cell membrane, preventing efficient Env clustering; a crucial step in initiating HIV-1 fusion (11). IFITMs have also been reported to restrict HIV-1 fusion and entry by modulation of the lipid bilayer (75). Biochemical studies have shown membrane fluidity influences HIV-1 Env mobility, stability, and conformation (76, 77). Importantly, Env clustering and mobility in lipid membranes is dependent on the EnvCT. Super-resolution microscopy of HIV-1 EnvCT Δ144 showed this mutant Env is highly mobile in the lipid bilayer and cannot cluster efficiently in membranes due to loss of interaction with the underlying Gag matrix lattice (21, 22). Thus, it is possible that increased mobility of Env in the viral membrane may also contribute to enabling ΔCT mutants to overcome both SERINC’s and IFITM’s restrictive effects that coalesce on a common mechanism for impairing virion fusion. While studying membrane dynamics in this context is challenging, whether this unifying mechanism explains evasion by these two groups of restriction factors warrants further investigation. Importantly, this does not exclude the combined effects of altered EnvCT mobility and altered conformation in regulating ΔCT evasion from restriction. In fact, it emphasizes the multifaceted nature of SERINC5 restriction mechanisms.

It is well established that HIV-1 viruses with a truncated EnvCT cannot replicate in T cells due to an Env incorporation defect (13–15). By contrast, we found that HIV-2 replicated efficiently in CD4+ H9 T cells without the EnvCT and showed no incorporation defect. These results are consistent with early observations that HIV-2 and several SIVs readily truncate the EnvCT in human PBMCs and T cell lines in vitro (23–31). This suggests that while a path to evasion of entry-targeting restriction factors through EnvCT truncation is possible, it is not equally available to both human lentiviruses, highlighting important differences in the requirement for the EnvCT between HIV-1 and HIV-2. Understanding how this works at the molecular level and why HIV-2 bypasses the need for the long EnvCT during viral assembly should be addressed in future work. We propose that further comparative analysis of HIV-1 and HIV-2 assembly may help explain the requirement of HIV-1 for the long EnvCT that has remained somewhat elusive and reveal important differences between successful and less-successful human lentiviruses. The rarity of HIV-2 infection makes it impossible to determine how frequently HIV-2 truncates the EnvCT in vivo, although a handful of cases are reported (24, 25, 28). It is intriguing that HIV-2 Nefs are less-potent antagonists of SERINC5 than HIV-1 and SIV_smm_ Nefs (35), and whether this reflects different pathways available to HIV-2 to evade SERINC5 restriction mediated by the EnvCT remains an open question. However, the observation that EnvCT truncation slows down the process of viral fusion suggests this would likely confer a fitness cost by increasing the window of opportunity for inhibition by neutralizing antibodies, as we have shown for HIV-1. Thus, even if HIV-2 could tolerate EnvCT truncation in vivo, this may potentially expose the virus to increased targeting by humoral immunity. This interplay between innate and adaptive immunity is exemplified by the observation that HIV-1 T/F viruses are resistant to IFITM restriction but become more sensitive over time when the selective pressure for chronic viruses to evade neutralizing antibodies becomes more dominant (43).

Clearly, viruses must navigate complex selective pressures from both the innate and adaptive immune system, while correctly orchestrating the essential steps of assembly and entry in order to transmit and mediate spreading infection. We propose that plasticity in HIV Env glycoproteins and their propensity to adopt various conformational states during viral entry affords a level of flexibility that may be exploited by the virus for this purpose and reveal a key role for the long lentiviral EnvCT in these processes.

## Methods

### Cells and Viral Constructs.

HEK293T and HeLa TZMbl cell lines were grown in Dulbecco’s modified eagle’s medium (Thermo-Fisher Scientific) supplemented with 10% fetal calf serum (FCS) and 100 U/mL penicillin–streptomycin. H9 cells were grown in Roswell Park Memorial Institute Medium 1640 (Thermo-Fisher Scientific) supplemented with 10% FCS and 100U/mL penicillin–streptomycin. Proviral constructs expressing full-length, replication-competent HIV-1 NL4.3, HIV-2 ST, and 7312A isolates were used (obtained from the Center for AIDS Reagents, UK). Site-directed mutagenesis was carried out using the QuikChange Lightening kit (Agilent) as per manufacturer’s instructions using primers listed in *SI Appendix*, *Supplementary Methods*. Viral stocks were generated by transfecting HEK293T cells using Fugene 6 (Promega). After 48 h, virus was harvested and titrated by infectivity assay on HeLa TZMbl reporter cells using Bright-Glo luciferase (Promega).

### SERINC Restriction Assay.

Plasmids encoding SERINC proteins HA-tagged at the N terminus (intracellular tag) and FLAG-tagged in the fourth extracellular loop were a gift from Massimo Pizzato, University of Trento, Italy. 293T cells seeded in 6-well plates (8 × 10^5^/well) were transfected with 600 ng proviral DNA, 0- to 200-ng titration of pSERINC3/5 DNA, and empty pcDNA vector to equalize DNA content. Transfected cells and virus-containing supernatants were collected at 48 h post-transfection and analyzed. Virus in supernatant was quantified by RT activity using SG-PERT assay (33). Virion infectivity was determined by luciferase assay using HeLa TZMbl reporter cells. Particle infectivity was calculated by normalizing infectivity RLU to RT activity as measured by SG-PERT and expressed as an RLU/RT ratio.

### Flow Cytometry.

HEK293T cells were washed and surface stained at 4 °C with primary antibodies for SERINC5 and cell viability dye Zombie UV (Biolegend). For intracellular staining, cells were fixed with 4% paraformaldehyde and permeabilized with CytoPerm buffer (Biolegend). Primary antibody anti-HA.11 epitope tag-PE (16B12, Biolegend) was used to detect SERINC5 and IFITMs. Data were acquired on a BD LSR Fortessa X-20 or Calibur cytometer and analyzed using FlowJo.

### Western Blotting.

Virions were purified by ultracentrifugation for 2 h over a 25% sucrose cushion. Cells and virus were lysed using 2x Laemmli buffer containing 50 mM Tris(2-carboxyethyl)phosphine (Sigma). Equal volumes of purified virus or cell lysates were heated at 37 °C for 1 h, separated by sodium dodecyl sulfate polyacrylamide gel electrophoresis, and analyzed by Western blotting using primary antibodies in *SI Appendix*, *Supplementary Methods*. Immunoblots were imaged by Odyssey Infrared Imager (Licor) and analyzed with Image Studio Lite software.

### Blam-Vpr Assay.

BLAM-Vpr–containing viruses were produced by cotransfecting HEK293T cells with pNL4.3 WT and ΔCT, pAdVantage, pBLAM-Vpr, and 100 ng pSERINC5. The assay was performed using the Invitrogen kit (K1085) as described (37).

### Co-IP Assay.

To investigate the interaction of SERINC5 and Env WT and ∆CT co-IP was performed as described in *SI Appendix*, *Supplementary Methods*.

### Neutralization Assays.

Neutralization assays were performed as described in *SI Appendix*, *Supplementary Methods*.

### T20 Chase Assay.

T20 chase assays were performed as described in *SI Appendix*, *Supplementary Methods*.

### Thermostability Assay.

Thermostability assays were performed as described in *SI Appendix*, *Supplementary Methods*.

### IFITM Restriction Assay.

HeLa TZMbl reporter cells were transduced with pSIN vectors expressing HA-tagged IFITM1, IFITM2, or IFITM3. Vectors were gifted by Greg Towers (University College London, United Kingdom). IFITM-expressing cells were selected using puromycin and expression confirmed by flow cytometry. Cells plated in a 96-well plate were infected with equal RT units of HIV-1 or HIV-2 viruses for 24 h and infection measured by luciferase assay.

### H9 Spreading Infections.

1 × 10^6^ H9 cells were infected with 10 mU/mL RT units of VSV-G pseudotyped virus for 4 h at 37 °C. Virus-containing supernatants were removed and cells were washed once in phosphate-buffered saline before being resuspended in fresh RPMI media. At sequential days postinfection, virus-containing supernatants were harvested, and viral content quantified by SG-PERT.

### Statistical Analysis.

Statistical significance was calculated using paired or unpaired Student’s *t* test or two-way ANOVA. Significance was assumed when *P* < 0.05. All statistical analyses were calculated using Prism 6 (GraphPad Prism).

## Supplementary Material

Supplementary File

## Data Availability

All study data are included in the article and/or *SI Appendix*.

## References

[1] R. P. Sumner., Are evolution and the intracellular innate immune system key determinants in HIV transmission? Front. Immunol. 8, 1246 (2017). 29056936 10.3389/fimmu.2017.01246 PMC5635324

[2] T. L. Foster, S. Pickering, S. J. D. Neil, Inhibiting the ins and outs of HIV replication: Cell-intrinsic antiretroviral restrictions at the plasma membrane. Front. Immunol. 8, 1853 (2018). 29354117 10.3389/fimmu.2017.01853 PMC5758531

[3] A. Rosa., HIV-1 Nef promotes infection by excluding SERINC5 from virion incorporation. Nature 526, 212–217 (2015). 26416734 10.1038/nature15399 PMC4861059

[4] Y. Usami, Y. Wu, H. G. Göttlinger, SERINC3 and SERINC5 restrict HIV-1 infectivity and are counteracted by Nef. Nature 526, 218–223 (2015). 26416733 10.1038/nature15400 PMC4600458

[5] A. Chande., S2 from equine infectious anemia virus is an infectivity factor which counteracts the retroviral inhibitors SERINC5 and SERINC3. Proc. Natl. Acad. Sci. U.S.A. 113, 13197–13202 (2016). 27803322 10.1073/pnas.1612044113 PMC5135340

[6] Y. S. Ahi., Functional interplay between murine leukemia virus glycogag, Serinc5, and surface glycoprotein governs virus entry, with opposite effects on gammaretroviral and ebolavirus glycoproteins. mBio 7, e01985-16 (2016). 10.1128/mBio.01985-16 PMC5120145 27879338

[7] V. E. Pye., A bipartite structural organization defines the SERINC family of HIV-1 restriction factors. Nat. Struct. Mol. Biol. 27, 78–83 (2020). 31907454 10.1038/s41594-019-0357-0 PMC6956856

[8] C. Sood, M. Marin, A. Chande, M. Pizzato, G. B. Melikyan, SERINC5 protein inhibits HIV-1 fusion pore formation by promoting functional inactivation of envelope glycoproteins. J. Biol. Chem. 292, 6014–6026 (2017). 28179429 10.1074/jbc.M117.777714 PMC5392591

[9] J. Shi., HIV-1 Nef antagonizes SERINC5 restriction by downregulation of SERINC5 via the endosome/lysosome system. J. Virol. 92, e00196-18 (2018). 29514909 10.1128/JVI.00196-18 PMC5952139

[10] S. Beitari, S. Ding, Q. Pan, A. Finzi, C. Liang, Effect of HIV-1 env on SERINC5 antagonism. J. Virol. 91, e02214-16 (2017). 27928004 10.1128/JVI.02214-16 PMC5286904

[11] Y. C. Chen., Super-resolution fluorescence imaging reveals that serine incorporator protein 5 inhibits human immunodeficiency virus fusion by disrupting envelope glycoprotein clusters. ACS Nano 14, 10929–10943 (2020). 32441921 10.1021/acsnano.0c02699 PMC8274448

[12] X. Zhang., CD4 expression and env conformation are critical for HIV-1 restriction by SERINC5. J. Virol. 93, e00544-19 (2019). 31043528 10.1128/JVI.00544-19 PMC6600204

[13] M. A. Checkley, B. G. Luttge, E. O. Freed, HIV-1 envelope glycoprotein biosynthesis, trafficking, and incorporation. J. Mol. Biol. 410, 582–608 (2011). 21762802 10.1016/j.jmb.2011.04.042 PMC3139147

[14] H. Akari, T. Fukumori, A. Adachi, Cell-dependent requirement of human immunodeficiency virus type 1 gp41 cytoplasmic tail for Env incorporation into virions. J. Virol. 74, 4891–4893 (2000). 10775630 10.1128/jvi.74.10.4891-4893.2000 PMC112014

[15] T. Murakami, E. O. Freed, The long cytoplasmic tail of gp41 is required in a cell type-dependent manner for HIV-1 envelope glycoprotein incorporation into virions. Proc. Natl. Acad. Sci. U.S.A. 97, 343–348 (2000). 10618420 10.1073/pnas.97.1.343 PMC26665

[16] C. Berlioz-Torrent., Interactions of the cytoplasmic domains of human and simian retroviral transmembrane proteins with components of the clathrin adaptor complexes modulate intracellular and cell surface expression of envelope glycoproteins. J. Virol. 73, 1350–1361 (1999). 9882340 10.1128/jvi.73.2.1350-1361.1999 PMC103959

[17] S. Wyss., The highly conserved C-terminal dileucine motif in the cytosolic domain of the human immunodeficiency virus type 1 envelope glycoprotein is critical for its association with the AP-1 clathrin adaptor [correction of adapter]. J. Virol. 75, 2982–2992 (2001). 11222723 10.1128/JVI.75.6.2982-2992.2001 PMC115924

[18] R. Byland, P. J. Vance, J. A. Hoxie, M. Marsh, A conserved dileucine motif mediates clathrin and AP-2-dependent endocytosis of the HIV-1 envelope protein. Mol. Biol. Cell 18, 414–425 (2007). 17108326 10.1091/mbc.E06-06-0535 PMC1783771

[19] M. Boge, S. Wyss, J. S. Bonifacino, M. Thali, A membrane-proximal tyrosine-based signal mediates internalization of the HIV-1 envelope glycoprotein via interaction with the AP-2 clathrin adaptor. J. Biol. Chem. 273, 15773–15778 (1998). 9624176 10.1074/jbc.273.25.15773

[20] R. E. Murphy, A. B. Samal, J. Vlach, J. S. Saad, Solution structure and membrane interaction of the cytoplasmic tail of HIV-1 gp41 protein. Structure 25, 1708–1718.e5 (2017). 29056482 10.1016/j.str.2017.09.010 PMC5687296

[21] W. Muranyi, S. Malkusch, B. Müller, M. Heilemann, H. G. Kräusslich, Super-resolution microscopy reveals specific recruitment of HIV-1 envelope proteins to viral assembly sites dependent on the envelope C-terminal tail. PLoS Pathog. 9, e1003198 (2013). 23468635 10.1371/journal.ppat.1003198 PMC3585150

[22] N. Pezeshkian, N. S. Groves, S. B. van Engelenburg, Single-molecule imaging of HIV-1 envelope glycoprotein dynamics and Gag lattice association exposes determinants responsible for virus incorporation. Proc. Natl. Acad. Sci. U.S.A. 116, 25269–25277 (2019). 31757854 10.1073/pnas.1910008116 PMC6911212

[23] T. Kodama., Significance of premature stop codons in env of simian immunodeficiency virus. J. Virol. 63, 4709–4714 (1989). 2795718 10.1128/jvi.63.11.4709-4714.1989 PMC251107

[24] L. A. Evans., Characterization of a noncytopathic HIV-2 strain with unusual effects on CD4 expression. Science 240, 1522–1525 (1988). 2836951 10.1126/science.2836951

[25] J. Albert., Isolation of human immunodeficiency virus (HIV) from plasma during primary HIV infection. J. Med. Virol. 23, 67–73 (1987). 3479517 10.1002/jmv.1890230108

[26] G. Franchini., Molecular and biological characterization of a replication competent human immunodeficiency type 2 (HIV-2) proviral clone. Proc. Natl. Acad. Sci. U.S.A. 86, 2433–2437 (1989). 2648404 10.1073/pnas.86.7.2433 PMC286927

[27] L. Chakrabarti., Sequence of simian immunodeficiency virus from macaque and its relationship to other human and simian retroviruses. Nature 328, 543–547 (1987). 3649576 10.1038/328543a0

[28] L. I. Kong., West African HIV-2-related human retrovirus with attenuated cytopathicity. Science 240, 1525–1529 (1988). 3375832 10.1126/science.3375832

[29] V. Hirsch, N. Riedel, J. I. Mullins, The genome organization of STLV-3 is similar to that of the AIDS virus except for a truncated transmembrane protein. Cell 49, 307–319 (1987). 3646094 10.1016/0092-8674(87)90283-2

[30] V. M. Hirsch., SIV adaptation to human cells. Nature 341, 573–574 (1989). 2677749 10.1038/341573a0

[31] V. M. Hirsch, R. A. Olmsted, M. Murphey-Corb, R. H. Purcell, P. R. Johnson, An African primate lentivirus (SIVsm) closely related to HIV-2. Nature 339, 389–392 (1989). 2786147 10.1038/339389a0

[32] E. O. Freed, M. A. Martin, Virion incorporation of envelope glycoproteins with long but not short cytoplasmic tails is blocked by specific, single amino acid substitutions in the human immunodeficiency virus type 1 matrix. J. Virol. 69, 1984–1989 (1995). 7853546 10.1128/jvi.69.3.1984-1989.1995 PMC188822

[33] M. Pizzato., A one-step SYBR Green I-based product-enhanced reverse transcriptase assay for the quantitation of retroviruses in cell culture supernatants. J. Virol. Methods 156, 1–7 (2009). 19022294 10.1016/j.jviromet.2008.10.012

[34] J. R. Day, C. Münk, J. C. Guatelli, The membrane-proximal tyrosine-based sorting signal of human immunodeficiency virus type 1 gp41 is required for optimal viral infectivity. J. Virol. 78, 1069–1079 (2004). 14722262 10.1128/JVI.78.3.1069-1079.2004 PMC321364

[35] A. Heigele., The potency of Nef-mediated SERINC5 antagonism correlates with the prevalence of primate lentiviruses in the wild. Cell Host Microbe 20, 381–391 (2016). 27631701 10.1016/j.chom.2016.08.004 PMC5098270

[36] A. E. Ward., HIV-cell membrane fusion intermediates are restricted by Serincs as revealed by cryo-electron and TIRF microscopy. J. Biol. Chem. 295, 15183–15195 (2020). 32788212 10.1074/jbc.RA120.014466 PMC7650252

[37] M. Cavrois, C. De Noronha, W. C. Greene, A sensitive and specific enzyme-based assay detecting HIV-1 virion fusion in primary T lymphocytes. Nat. Biotechnol. 20, 1151–1154 (2002). 12355096 10.1038/nbt745

[38] T. Zhou., Structural basis for broad and potent neutralization of HIV-1 by antibody VRC01. Science 329, 811–817 (2010). 20616231 10.1126/science.1192819 PMC2981354

[39] E. Falkowska., Broadly neutralizing HIV antibodies define a glycan-dependent epitope on the prefusion conformation of gp41 on cleaved envelope trimers. Immunity 40, 657–668 (2014). 24768347 10.1016/j.immuni.2014.04.009 PMC4070425

[40] N. Sullivan., CD4-Induced conformational changes in the human immunodeficiency virus type 1 gp120 glycoprotein: Consequences for virus entry and neutralization. J. Virol. 72, 4694–4703 (1998). 9573233 10.1128/jvi.72.6.4694-4703.1998 PMC109994

[41] J. Lu., The IFITM proteins inhibit HIV-1 infection. J. Virol. 85, 2126–2137 (2011). Corrected in: *J. Virol.* **85**, 4043 (2011). 21177806 10.1128/JVI.01531-10 PMC3067758

[42] J. Qian., Primate lentiviruses are differentially inhibited by interferon-induced transmembrane proteins. Virology 474, 10–18 (2015). 25463599 10.1016/j.virol.2014.10.015 PMC4581848

[43] T. L. Foster., Resistance of transmitted founder HIV-1 to IFITM-mediated restriction. Cell Host Microbe 20, 429–442 (2016). 27640936 10.1016/j.chom.2016.08.006 PMC5075283

[44] M. OhAinle., A virus-packageable CRISPR screen identifies host factors mediating interferon inhibition of HIV. eLife 7, e39823 (2018). 30520725 10.7554/eLife.39823 PMC6286125

[45] A. A. Compton., IFITM proteins incorporated into HIV-1 virions impair viral fusion and spread. Cell Host Microbe 16, 736–747 (2014). 25464829 10.1016/j.chom.2014.11.001 PMC7104936

[46] K. Tartour., IFITM proteins are incorporated onto HIV-1 virion particles and negatively imprint their infectivity. Retrovirology 11, 103 (2014). 25422070 10.1186/s12977-014-0103-y PMC4251951

[47] J. Yu., IFITM proteins restrict HIV-1 infection by antagonizing the envelope glycoprotein. Cell Rep. 13, 145–156 (2015). 26387945 10.1016/j.celrep.2015.08.055 PMC4602366

[48] D. A. Lazzarino, M. Diego, E. Musi, S. Z. Hirschman, R. J. Alexander, CXCR4 and CCR5 expression by H9 T-cells is downregulated by a peptide-nucleic acid immunomodulator. Immunol. Lett. 74, 189–195 (2000). 11064099 10.1016/s0165-2478(00)00258-3

[49] Q. Wang, A. Finzi, J. Sodroski, The conformational states of the HIV-1 envelope glycoproteins. Trends Microbiol. 28, 655–667 (2020). 32418859 10.1016/j.tim.2020.03.007 PMC7363548

[50] A. Bartesaghi, A. Merk, M. J. Borgnia, J. L. Milne, S. Subramaniam, Prefusion structure of trimeric HIV-1 envelope glycoprotein determined by cryo-electron microscopy. Nat. Struct. Mol. Biol. 20, 1352–1357 (2013). 24154805 10.1038/nsmb.2711 PMC3917492

[51] M. Pancera., Structure and immune recognition of trimeric pre-fusion HIV-1 Env. Nature 514, 455–461 (2014). 25296255 10.1038/nature13808 PMC4348022

[52] D. Lyumkis., Cryo-EM structure of a fully glycosylated soluble cleaved HIV-1 envelope trimer. Science 342, 1484–1490 (2013). 24179160 10.1126/science.1245627 PMC3954647

[53] G. Ozorowski., Open and closed structures reveal allostery and pliability in the HIV-1 envelope spike. Nature 547, 360–363 (2017). 28700571 10.1038/nature23010 PMC5538736

[54] G. Frey., A fusion-intermediate state of HIV-1 gp41 targeted by broadly neutralizing antibodies. Proc. Natl. Acad. Sci. U.S.A. 105, 3739–3744 (2008). 18322015 10.1073/pnas.0800255105 PMC2268799

[55] A. Piai., Structural basis of transmembrane coupling of the HIV-1 envelope glycoprotein. Nat. Commun. 11, 2317 (2020). 32385256 10.1038/s41467-020-16165-0 PMC7210310

[56] E. O. Freed, M. A. Martin, Domains of the human immunodeficiency virus type 1 matrix and gp41 cytoplasmic tail required for envelope incorporation into virions. J. Virol. 70, 341–351 (1996). 8523546 10.1128/jvi.70.1.341-351.1996 PMC189823

[57] T. G. Edwards., Relationships between CD4 independence, neutralization sensitivity, and exposure of a CD4-induced epitope in a human immunodeficiency virus type 1 envelope protein. J. Virol. 75, 5230–5239 (2001). 11333905 10.1128/JVI.75.11.5230-5239.2001 PMC114929

[58] T. G. Edwards., Truncation of the cytoplasmic domain induces exposure of conserved regions in the ectodomain of human immunodeficiency virus type 1 envelope protein. J. Virol. 76, 2683–2691 (2002). 11861835 10.1128/JVI.76.6.2683-2691.2002 PMC135967

[59] J. Chen., HIV-1 ENVELOPE. Effect of the cytoplasmic domain on antigenic characteristics of HIV-1 envelope glycoprotein. Science 349, 191–195 (2015). 26113642 10.1126/science.aaa9804 PMC4701381

[60] E. White., Truncating the gp41 cytoplasmic tail of simian immunodeficiency virus decreases sensitivity to neutralizing antibodies without increasing the envelope content of virions. J. Virol. 92, e01688-17 (2018). 29142124 10.1128/JVI.01688-17 PMC5774881

[61] A. N. Vzorov, K. M. Gernert, R. W. Compans, Multiple domains of the SIV Env protein determine virus replication efficiency and neutralization sensitivity. Virology 332, 89–101 (2005). 15661143 10.1016/j.virol.2004.10.044

[62] S. Wyss., Regulation of human immunodeficiency virus type 1 envelope glycoprotein fusion by a membrane-interactive domain in the gp41 cytoplasmic tail. J. Virol. 79, 12231–12241 (2005). 16160149 10.1128/JVI.79.19.12231-12241.2005 PMC1211532

[63] T. Kuwata, T. Kaori, I. Enomoto, K. Yoshimura, S. Matsushita, Increased infectivity in human cells and resistance to antibody-mediated neutralization by truncation of the SIV gp41 cytoplasmic tail. Front. Microbiol. 4, 117 (2013). 23717307 10.3389/fmicb.2013.00117 PMC3653066

[64] L. G. Abrahamyan., The cytoplasmic tail slows the folding of human immunodeficiency virus type 1 Env from a late prebundle configuration into the six-helix bundle. J. Virol. 79, 106–115 (2005). 15596806 10.1128/JVI.79.1.106-115.2005 PMC538707

[65] J. W. Dubay, S. R. Dubay, H. J. Shin, E. Hunter, Analysis of the cleavage site of the human immunodeficiency virus type 1 glycoprotein: Requirement of precursor cleavage for glycoprotein incorporation. J. Virol. 69, 4675–4682 (1995). 7609032 10.1128/jvi.69.8.4675-4682.1995 PMC189271

[66] P. B. Johnston, J. W. Dubay, E. Hunter, Truncations of the simian immunodeficiency virus transmembrane protein confer expanded virus host range by removing a block to virus entry into cells. J. Virol. 67, 3077–3086 (1993). 8497044 10.1128/jvi.67.6.3077-3086.1993 PMC237644

[67] G. D. Ritter, Jr, M. J. Mulligan, S. L. Lydy, R. W. Compans, Cell fusion activity of the simian immunodeficiency virus envelope protein is modulated by the intracytoplasmic domain. Virology 197, 255–264 (1993). 8212561 10.1006/viro.1993.1586

[68] N. D. Durham., Neutralization resistance of virological synapse-mediated HIV-1 Infection is regulated by the gp41 cytoplasmic tail. J. Virol. 86, 7484–7495 (2012). 22553332 10.1128/JVI.00230-12 PMC3416307

[69] B. A. Puffer., CD4 independence of simian immunodeficiency virus Envs is associated with macrophage tropism, neutralization sensitivity, and attenuated pathogenicity. J. Virol. 76, 2595–2605 (2002). 11861825 10.1128/JVI.76.6.2595-2605.2002 PMC135960

[70] E. Yuste, W. Johnson, G. N. Pavlakis, R. C. Desrosiers, Virion envelope content, infectivity, and neutralization sensitivity of simian immunodeficiency virus. J. Virol. 79, 12455–12463 (2005). 16160173 10.1128/JVI.79.19.12455-12463.2005 PMC1211544

[71] Y. Wang., The V3 loop of HIV-1 env determines viral susceptibility to IFITM3 impairment of viral infectivity. J. Virol. 91, e02441-16 (2017). 28100616 10.1128/JVI.02441-16 PMC5355610

[72] W. L. Wu., Δ20 IFITM2 differentially restricts X4 and R5 HIV-1. Proc. Natl. Acad. Sci. U.S.A. 114, 7112–7117 (2017). 28630320 10.1073/pnas.1619640114 PMC5502592

[73] J. Yu, S. L. Liu, The inhibition of HIV-1 entry imposed by interferon inducible transmembrane proteins is independent of co-receptor usage. Viruses 10, 413 (2018). 10.3390/v10080413 PMC6115839 30087232

[74] S. Ding, Q. Pan, S. L. Liu, C. Liang, HIV-1 mutates to evade IFITM1 restriction. Virology 454-455, 11–24 (2014). 24725927 10.1016/j.virol.2014.01.020 PMC4274668

[75] K. Li., IFITM proteins restrict viral membrane hemifusion. PLoS Pathog. 9, e1003124 (2013). 23358889 10.1371/journal.ppat.1003124 PMC3554583

[76] H. Salimi., The lipid membrane of HIV-1 stabilizes the viral envelope glycoproteins and modulates their sensitivity to antibody neutralization. J. Biol. Chem. 295, 348–362 (2020). 31757809 10.1074/jbc.RA119.009481 PMC6956520

[77] J. A. Nieto-Garai., Cholesterol in the viral membrane is a molecular switch governing HIV-1 env clustering. Adv. Sci. (Weinh.) 8, 2003468 (2020). 33552873 10.1002/advs.202003468 PMC7856888

