# Supplementary material for: HIV envelope tail truncation confers resistance to SERINC5 restriction
Source: Proc Natl Acad Sci U S A. Author manuscript; Available in PMC 2021 Jun 9. (PMC8166163; doi:10.1073/pnas.2101450118)
Supplement: SI [file EMS125793-supplement-SI.pdf]

PNAS
www.pnas.org

Supplementary Information for
HIV Envelope tail truncation confers resistance to SERINC5 restriction

Tafhima Haider, Xenia Snetkov and Clare Jolly*

Clare Jolly
Email: c.jolly@ucl.ac.uk

This PDF file includes:

Supplementary text including methods
Figures S1 to S4
SI References

Supplementary Information Text
Supplementary Methods:

Antibodies used for western blotting:

Anti-HIV-1 gp120 rabbit antisera (donated by Dr S. Ranjibar and obtained from the CFAR); anti-HIV-1 Gag rabbit antisera (donated by DR G. Reid and obtained from the CFAR); Anti-HIV-1 p24 Monoclonal 183-H12-5C (donated by Dr. Bruce Chesebro and Kathy Wehrly and obtained from CFAR); anti-HA.11 epitope tag-PE (16B12, Biolegend) and anti-tubulin (Sigma). Primary antibodies were detected with appropriate fluorescent secondary antibodies: anti-Rabbit IgG (ab216773, Abcam), anti-Mouse IgG (ab216775, Abcam) and anti-Human IgG (926-32232, Licor).

Coimmunoprecipitation assay.

5 x 10^6 HEK293T cells were co-transfected with 4ug pNL4.3 (ΔEnv, WT or ΔCT) and 1ug pcDNA-flag-SERINC5-HA using Fusion 6 transfection reagent (Promega). 48hr post transfection, culture media was removed, and cells were washed once in PBS and lysed in ice cold Pierce IP lysis buffer (Thermo scientific). The soluble fraction was recovered and incubated with anti-FLAG M2 magnetic beads (Sigma) overnight at 4°C to pulldown flag-SERINC5-HA. FLAG beads were magnetically separated from the lysate and washed four times with ice cold IP buffer. Flag-SERINC5-HA was eluted from FLAG beads using 50μL of 3x-FLAG peptide (Sigma) in IP buffer (500μg/ml) for 30mins rotating at 4°C. The input cell lysate and eluted samples were prepared for immunoblot analysis as described above. Flag-SERINC5-HA was detected using anti-HA.11 epitope tag-PE (16B12, Biolegend) and Env detected by anti-HIV-1 gp120 rabbit antisera. Transferrin receptor (M-A712, BD Biosciences) was used as a cellular membrane protein control and Gag (p24) as a viral protein control.

Neutralisation assays

Virus supernatants were incubated with serial dilutions of VRC01 (1), PGT151 (2) 2F5 (3), 10E8 (4), 17b (5), sCD4 (Progenics pharmaceuticals) or T20 fusion inhibitor (DAIDS, NIAID) and incubated at 37 °C for 1 h (all antibodies from CFAR except PGT151 which was a gift from Laura McCoy, UCL). An additional 1 h preincubation with sCD4 was performed prior to incubation with 17b for indicated experiments. Next, virus-inhibitor mixtures were transferred to HeLa TZMbl reporter cells and luciferase activity was measured after 48h using Bright-Glo. Antibody neutralisation was calculated as percent decrease in luciferase activity compared to the corresponding virus-only control. IC50 values were calculated by nonlinear regression analysis (sigmoid curve interpolation) using Prism software (GraphPad).

T20 chase assay

Equal RT units of virus were added to TZMbl cells and 20 μg/ml T20 inhibitor was added at 0, 1, 2, 4, 6 and 24h post-addition of virus. Viral infection was quantified after 48h by measuring luciferase activity. The percentage viral entry was determined by normalising infectivity in at each time point to the untreated (no T20) control from three independent experiments.

Thermostability assay

At indicated time points, viruses were thawed and incubated at 37°C for different durations. After 6 hours, freshly thawed virus was used to infect TZMbl cells (t=0 h) and previously incubated virus were also used to infect cells. Cells were lysed after 48h in BrightGlo and luciferase measured.

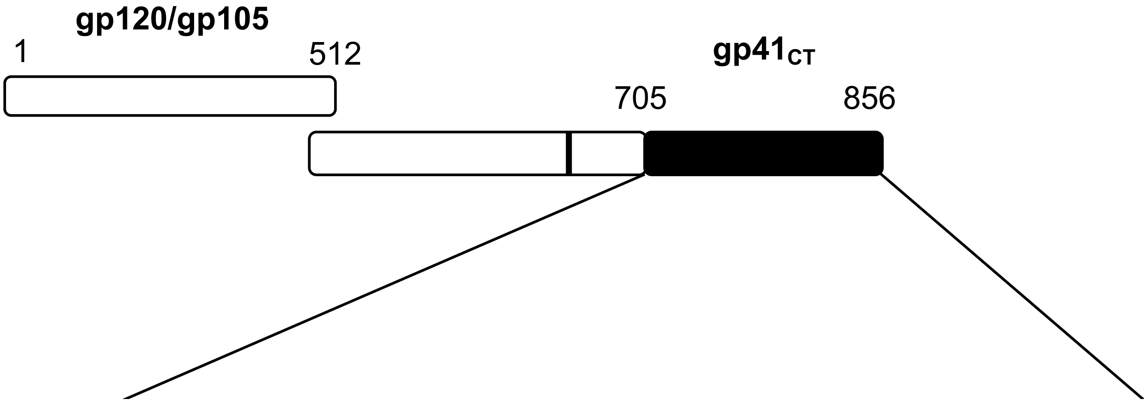

Virus	Mutations in EnvCT	
HIV-1 NL4.3	YSPLSFQTHLPI*RGPDRPEGIEEEGGERDRDRSIRLVNGSLALIWDDLRSLCLFSYHRL<br>RDLLLIVTRIVELLGRRGWEALKYWWNLLQYWSQELKNSAVNLLNATAIAVAEGTDRVIE<br>VLQAAYRAIRHIPRRIRQGLERILL*	P722stop
HIV-2 7312A	YRPVFSSPPAYF*QIHIHKDREQPAREETEEDVGNSVGDNWWPWPIRYIHFLIRQLIRLL<br>NRLYNICRDLLSRSFQTLQLISQSLRRALTAVRDWLRFNTAYLQYGGEWIQEAFRAFARA<br>TGETLTNAWRGFWGTLGQIGRGILAVPRRIRQGAEIALL*	Q713stop
HIV-2 ST	YRPVFSSPPAYF*QIHIHKDREQPAREETEEDVGNSVGDNWWPWPIRYIHFLIRQLIRLL<br>NRLYNICRDLLSRSFQTLQLISQSLRRALTAVRDWLRFNTAYLQYGGEWIQEAFRAFARA<br>TGETLTNAWRGFWGTLGQIGRGILAVPRRIRQGAEIALL*	Q713stop

Supplementary Figure 1: Schematic of HIV EnvCT. EnvCT amino acid sequences of HIV-1 NL4.3 (Accession number: MN685337), HIV-2 7312A (Accession number: L36874) and HIV-2 ST (Accession number: M31113) are shown with the conserved endocytic motif underlined and insertion of a stop codon indicated by an *.

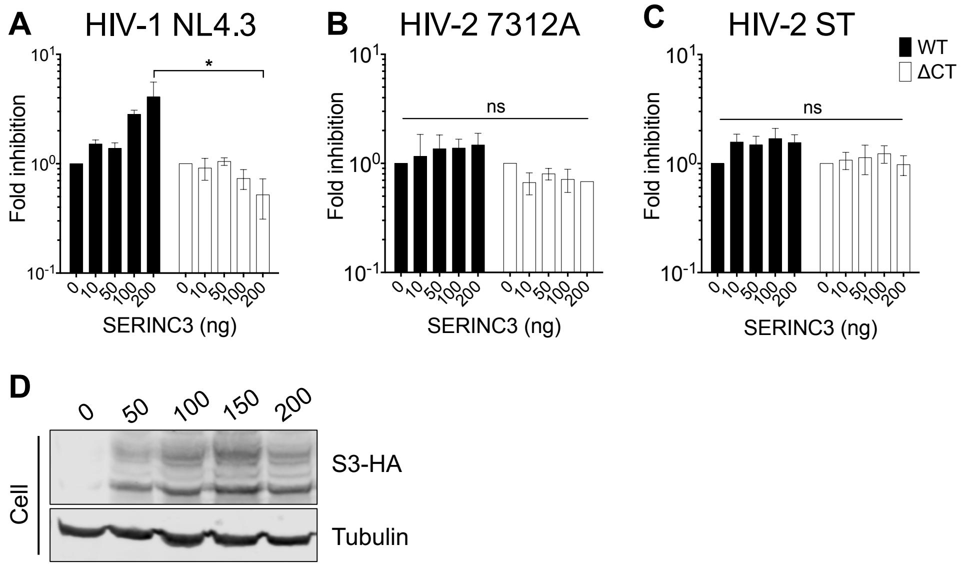

Supplementary Figure 2: Truncating the HIV EnvCT confers resistance to SERINC3 restriction. 293T cells were cotransfected with molecular clones encoding full length HIV-1 NL4.3, HIV-2 7312A and HIV-2 ST WT and ΔCT virus alongside indicated doses of pcDNA-based SERINC3 plasmid (Flag-SERINC3-HA). Virus containing supernatant were harvested after 48h. Budding was measured by quantifying RT activity in supernatants by SG-PERT assay. Infectivity was measured by titrating supernatant on to HeLa TZMbl reporter cells and measuring luciferase activity (RLU). Particle infectivity was calculated by normalising infectivity RLU to RT activity. **(A-C)** Fold inhibition of viral infectivity was calculated by normalising RLU/RT measurements to 0ng SERINC5. Bar graphs represent fold inhibition from three independent experiments. **(D)** Representative immunoblot of transfected 293T cell lysates confirming SERINC3 overexpression detected using antibody against HA-tagged SERINC3 (S3-HA). Bars show mean and error bars represent mean +/- SEM. The ΔCT fold inhibition was compared to WT fold inhibition at each dose of SERINC3 using two-tailed unpaired *t*-test (ns, P>0.05; *, P<0.05).

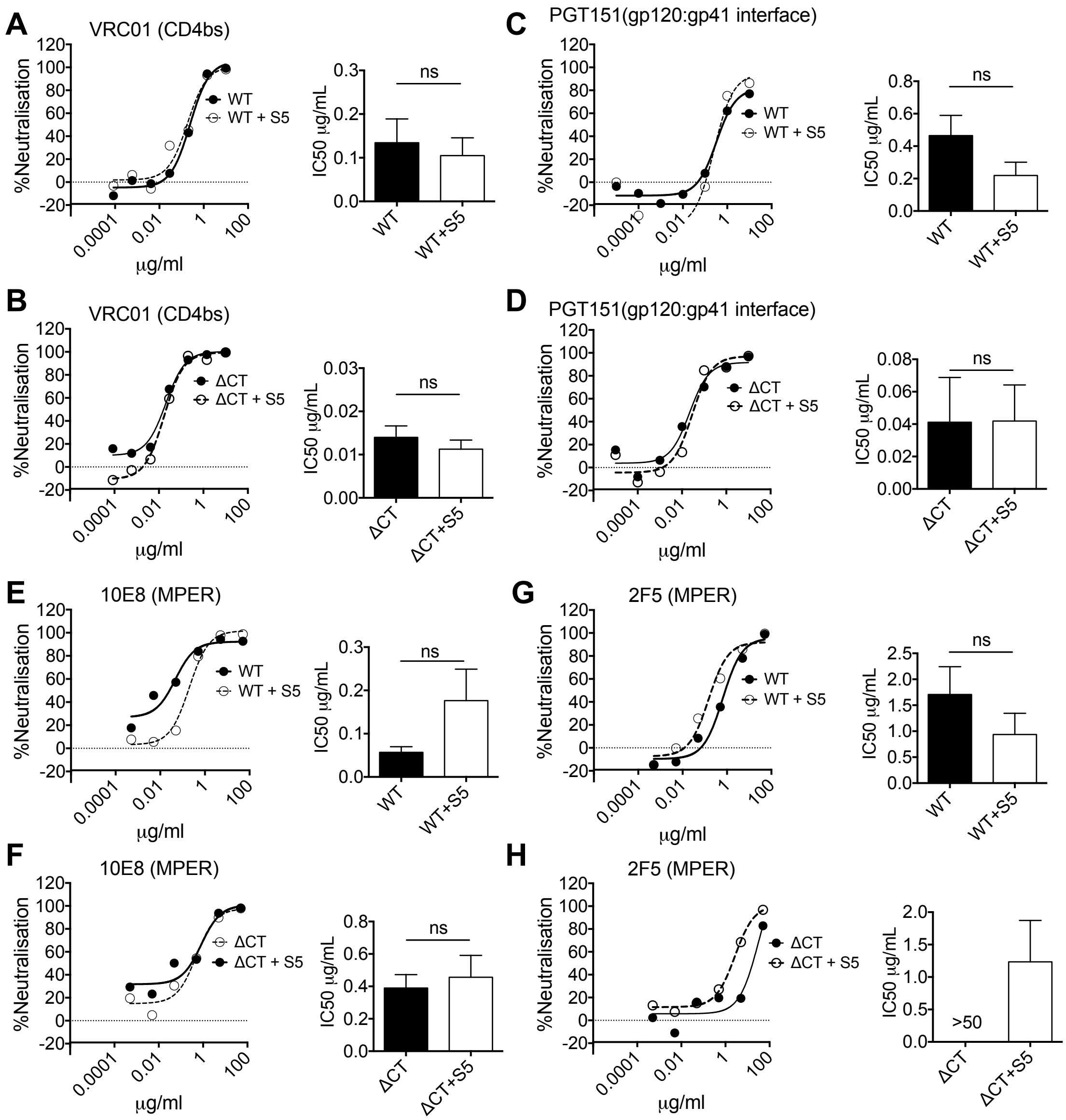

Supplementary Figure 3: Effect of SERINC5 incorporation on HIV-1 WT and ΔCT neutralisation profiles. (A-H). Neutralisation of HIV-1 WT and ΔCT viruses in the presence (solid lines, black symbols) and absence (dotted lines, white symbols) of SERINC5 in virus particles by broadly neutralising antibodies. Representative neutralisation curves are shown. Bar charts show IC50 values from three independent experiments. Bars represent mean and error bars represent SEM. Groups were compared using two-tailed unpaired *t*-test (ns, $p>0.05$).

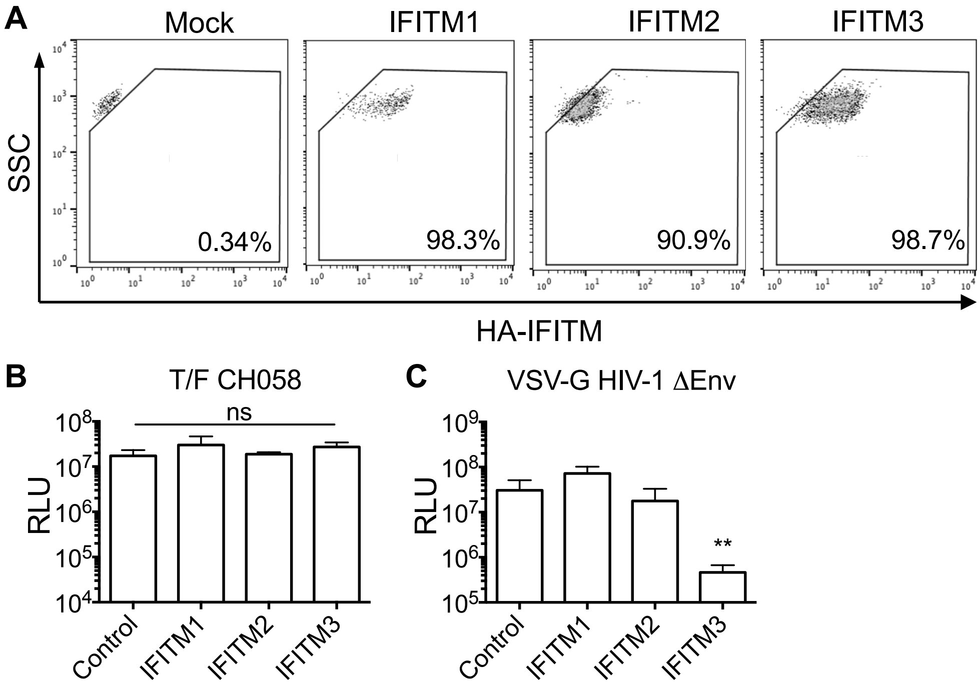

Supplementary Figure 4: Characterisation of IFITM overexpressing HeLa TZMbl cells. TZMbl cells were transduced with pSIN vectors expressing IFITM1, IFITM2 or IFITM3 and selected using puromycin. **(A)** Flow cytometry plots confirm expression of HA-tagged IFITM proteins in TZMbl cells after selection. Control TZMbl cells (no IFITM overexpression) and IFITM overexpressing TZMbl cells were infected with equal RT units of **(B)** T/F virus CH058 and **(C)** VSV-G pseudotyped HIV-1 ΔEnv virus virus for 24 h. Infectivity was measured using the luciferase assay, and data are from three independent experiments. Bars show mean and error bars represent mean +/- SEM from three independent experiments. Infectivity inhibition was compared using 2-way ANOVA test (ns, $p>0.05$; **, $p<0.01$).

SI References

1. T. Zhou *et al.*, Structural basis for broad and potent neutralization of HIV-1 by antibody VRC01. *Science* **329**, 811-817 (2010).
2. E. Falkowska *et al.*, Broadly neutralizing HIV antibodies define a glycan-dependent epitope on the prefusion conformation of gp41 on cleaved envelope trimers. *Immunity* **40**, 657-668 (2014).
3. T. Muster *et al.*, A conserved neutralizing epitope on gp41 of human immunodeficiency virus type 1. *J Virol* **67**, 6642-6647 (1993).
4. J. Huang *et al.*, Broad and potent neutralization of HIV-1 by a gp41-specific human antibody. *Nature* **491**, 406-412 (2012).
5. M. Thali *et al.*, Characterization of conserved human immunodeficiency virus type 1 gp120 neutralization epitopes exposed upon gp120-CD4 binding. *J Virol* **67**, 3978-3988 (1993).
